# Effects of methimepip and JNJ-5207852 in Wistar rats exposed to an open-field with and without object and in Balb/c mice exposed to a radial-arm maze

**DOI:** 10.3389/fnsys.2012.00054

**Published:** 2012-07-16

**Authors:** Rushdie M. A. Abuhamdah, Ruan van Rensburg, Natasha L. Lethbridge, Abdel Ennaceur, Paul L. Chazot

**Affiliations:** ^1^School of Biological and Biomedical Sciences, Durham UniversityDurham, UK; ^2^Leibniz Institute of Age Research, Fritz Lipmann InstituteJena, Germany; ^3^Sunderland Pharmacy School, University of SunderlandSunderland, UK

**Keywords:** fear, avoidance, anxiety, 3D maze, object novelty, histamine

## Abstract

The role of the histamine H_3_ receptor (H_3_R) in anxiety is controversial, due to limitations in drug selectivity and limited validity of behavioral tests used in previous studies. In the present report, we describe two experiments. In the first one, Wistar rats were treated with an H_3_R agonist (methimepip), and exposed to an open-field. In the second one, Balb/c mice were treated with H_3_R agonist (methimepip) or antagonist (JNJ-5207852), and exposed to an open space 3D maze which is a modified version of the radial-arm maze. C57BL/6J saline treated mice were included for comparisons. When exposed to an empty open field, Wistar rats spent more time in the outer area and made very low number of brief crossings in the central area. However, when an object occupied the central area, rats crossed frequently into and spent a long time in the central area. Administration of a range of different doses of methimepip (selective H_3_R agonist) reduced the entries into the central area with a novel object, indicating enhanced avoidance response. In the 3D maze, both Balb/c and C57BL/6J saline-treated mice crossed frequently onto the bridges that radiate from the central platform but only C57BL/6J mice crossed onto the arms which extend the bridges. This suggests that Balb/c mice are more anxious than C57BL/6J mice. Neither methimepip nor JNJ-5207852 (selective H_3_R antagonist/inverse agonist) induced entry into the arms of the maze, indicative of lack of anxiolytic effects.

## Introduction

The biogenic amine histamine is an important neurotransmitter and neuromodulator in the CNS that has been implicated in a variety of biological functions including thermo- and immunoregulation, food intake, hyperexcitability, pain transmission, arousal, reward, memory and emotional responses. The histamine H_3_ receptor (H_3_R) has been characterized as a presynaptic auto- and hetero-receptor on histaminergic and non-histaminergic neurons, respectively. It modulates histamine synthesis and release through negative feedback (Arrang et al., 1987; Giannoni et al., 2009). The basic organization and functional disposition of the histaminergic system is highly conserved in the vertebrate brain. In the mammalian brain histamine is synthesized and stored in the cell somata and axon varicosities in restricted populations of neurons that originate from the tuberomammillary nucleus (TMN) located in the posterior hypothalamus. Histaminergic neurons projecting from the ventral ascending pathway have strong innervation at the hypothalamus, diagonal band, septum, and olfactory bulb whilst the dorsal pathway has lower density fibers which innervate the thalamus, hippocampus, amygdala, and rostral forebrain structures, many of which play a role in cognitive and emotional behaviors (Haas et al., 2008). They also modulate the release of other neurotransmitters, including acetylcholine, norepinephrine, serotonin, and dopamine, implicated further in emotion (Arrang et al., 1995; Blandina et al., 1996; Threlfell et al., 2004; Haas et al., 2008; Giannoni et al., 2009). Preclinical studies suggest a role of H_3_Rs in a variety of cognitive disorders including attention deficit hyperactivity disorder, Alzheimer's disease and schizophrenia (see Esbenshade et al., 2008; Chazot, 2010; Leurs et al., 2011). There is growing evidence for a role of H_3_Rs in fear-avoidance (e.g., Baldi et al., 2005). However, a limited number of studies have investigated the role of H_3_Rs in anxiety, and these have proved inconsistent and even contradictory (Imaizumi and Onodera, 1993; Frisch et al., 1998; Pérez-García et al., 1999; Bongers et al., 2004; Dere et al., 2004; Rizk et al., 2004; Acevedo et al., 2006; Yokoyama et al., 2009). Both H_3_R agonists and antagonists were reported to produce anxiolysis, anxiogenesis or no effects in the current unconditioned tests of anxiety (Imaizumi and Onodera, 1993; Pérez-García et al., 1999; Rizk et al., 2004; Yokoyama et al., 2009). This is likely due to the selectivity of the compounds used in the older studies and/or limitations of the behavioral tests used for anxiety which all provides an option for escape/avoidance and, therefore, cannot distinguish between fear-induced avoidance and fear-induced anxiety (discussed in detail in Ennaceur, 2012).

It has been reported that rats and mice exposed to an open-field avoid the central area and avoid the presence of an object in this area (Hughes, 2007). It has been also reported that these animals avoid the open arms of the plus-maze (Handley and Mithani, 1984; Pellow et al., 1985) and the lit chamber of the light-dark box (Malin, 1974; Morgan and Kamp, 1980; Crawley, 1981). This avoidance behavior has been interpreted as an indicator of anxiety (discussed in Ennaceur et al., 2009b) though one cannot exclude the possibility that animals express a natural preference for protected and/or unlit spaces (see Malin, 1974; Morgan and Kamp, 1980; Buhot, 1989) and they may have no interest or motivation to venture into unprotected and/or lit spaces. Animals may also express fear from novelty and escape to or avoid from the protected and/or unlit space. In this case too, there is no objective evidence that demonstrate the interest or motivation of animals to approach the source of potential threat.

In the present report, we examined the behavior of Wistar male rats in the presence or absence of an object in the open-field, and we assessed whether this would be affected by methimepip, a selective histamine H_3_R agonist (Kitbunnadaj et al., 2005). As stated above, we believe that exposure for the first time to the open-field provides measures of fear-induced avoidance and we expect that methimepip would affect these measures. We also examined the behavior of Balb/c mice which were exposed to a 3D-maze and treated with or methimepip or JNJ-5207852, the latter, a selective histamine H_3_R antagonist (Barbier et al., 2004). In this second experiment, we included a group of C57BL/6J mice that were treated with saline for comparisons. The 3D maze is a modified version of the radial-arm maze. It consists of 8 arms attached to bridges that radiate from a central platform. Animals need to cross a bridge to access an arm of the maze. In this test apparatus C57BL/6J mice cross onto arms of the maze on first exposure (low anxiety strain), while Balb/c mice cross only onto the bridges (high anxiety strain), indicative of differential anxiety responses (Ennaceur et al., 2006, 2008; Ennaceur, 2011). If treatments in Balb/c mice induced crossing onto arms of the maze as seen with the control C57BL/6J mice, this would indicate an anxiolytic effect. This test has been validated previously with an anxiolytic agent, diazepam (Ennaceur et al., 2008).

The first experiment used rats because previous experiments in the open-field with and without object were conducted with these animals and demonstrated that the tests provide measures of fear-induced avoidance rather than fear-induced anxiety (Ennaceur et al., 2009a,b). When exposed for the first time to an open-field, rats show natural preference for the walls and corners and avoid the center of the field because there is nothing there to explore. When an object is present in the center they do approach and explore the object.

The second experiment used mice because these were previously assessed for anxiety in the 3D maze and differences in anxiety between strains of mice in this test is well established (Ennaceur et al., 2006, 2008). We have only experience with Wistar rats in this test but not with other strains of rats. It was not worthwhile testing mice in the open-field as in our view it does not provide measures of anxiety.

## Experiment 1—open-field with and without object

### Materials and methods

#### Animals

Forty nine male Wistar rats (six groups) supplied by Charles River Laboratories (Kent, UK) were used in the present study. The animal weight was 190–210 g at the time of the. The colony room was held under a 12 h light/12 h dark cycle (light 0700–1900 h at 180 Lux) and at 23°C ± 1°C. In order to avoid unequal light exposure, the upper shelf was occupied with plastic cages filled with sawdust. Rats were housed four per cage. Individual rats could be identified by their cage number and their color code created with indelible pen marker on their tail. Rats were left to acclimatize for 2 weeks before the start of the experiment. All rats had *ad libitum* access to food and water. During their stay in respective housing conditions, they were removed three times a week from their cages for cleaning the cages and renewing their food and water supply. Animal treatment and husbandry were in accordance with approved use of animals in scientific procedures regulated by the Animals (Scientific Procedures) Act 1986, UK.

#### Drug treatments

Two groups of male Wistar rats received physiological saline, the other groups received a single injection of one dose of methimepip (1 and 2.5 mg/kg i.p.) 30 min before exposure to the open-field with and without an object (Table 1). Methimepip was a kind gift obtained from Professor Rob Leurs (VU, Amsterdam, The Netherlands). Selection of dose concentrations was based on previous *in vivo* studies (e.g., Kitbunnadaj et al., 2005).

**Table 1:** **Groups and number of animals per groups in each test condition**.

	**Saline**	**Methimepip 1 mg/kg i.p.**	**Methimepip 2.5 mg/kg i.p.**
Open-field without object	*n* = 10	*n* = 7	*n* = 7
Open-field with object	*n* = 9	*n* = 8	*n* = 8

The drugs were administered i.p. 30 min before the test.

#### Apparatus and testing procedure

The apparatus consists of an open box (width 80 × length 80 × height 50 cm) made of gray PVC. The surface of the open-field was divided into outer, inner, and central areas. Each area was 16 cm wide. The illumination on the floor of the box apparatus was 186Lux. The objects (width 8 × length 8 × height 13 cm) to be explored were identical triplicate and were alternated between animals. They were made of white ceramic. Rats were released from the outer area of the open-field with the head oriented toward a wall. They were left to explore for 10 min.

#### Tools and recording measures

All sessions were video recorded and the behavior of rats was analyzed with an in-house computer program, EventLog. The recording of the behavior of rats was based on entries into defined areas of the apparatus. An entry was recorded whenever a rat crosses with all four paws into an area. EventLog records in sequential order the start and end of each crossing into an area of the open-field. It provides measures of latency, frequency, and duration of entries.

#### Measurement and statistical analysis

All data are expressed as mean ± s.e.m. Differences among group means values for each measurement were tested for significance with Two-Way ANOVA followed up with Newman–Keuls *post-hoc* comparisons (Statistica for Windows, version 5.5). Results are considered significant when *p* ≤ 0.05.

### Results

There were significant differences between groups [*F*_(2, 43)_ = 3.30, *p* < 0.05] and between test conditions [*F*_(1, 43)_ = 5.49, *p* < 0.02] in all test parameters except for latencies of entries into the inner and central areas (*p* > 0.10), and for the number of entries into the outer area (*p* > 0.10). There were, however, significant interactions between groups and test conditions only for the number of entries into the central area [*F*_(2, 43)_ = 9.37, *p* < 0.0004].

**Figure 1:**
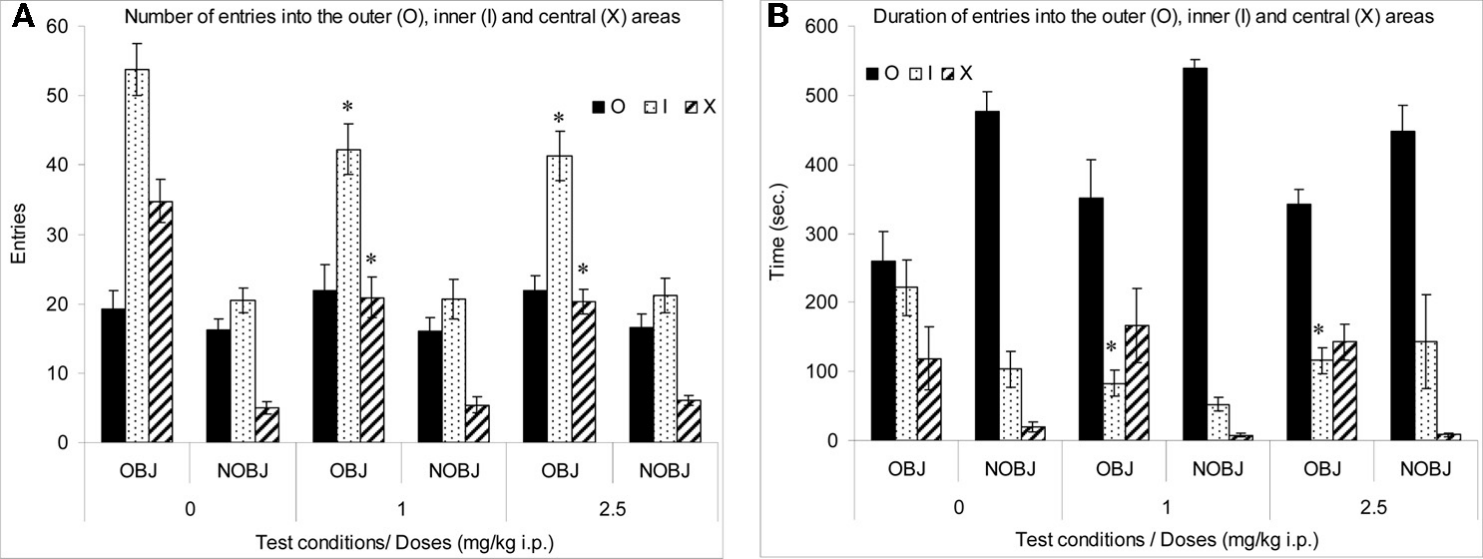
**Methimepip experiment with rats.** Mean (± s.e.m.) in the open-field with (OBJ) and without object (NOBJ). **(A)** Number of entries: ^*^compared to control in inner area (I), *p* < 0.05 and ^*^compared to control in central area for both doses of methimepip, *p* < 0.0002; **(B)** Duration of entries: ^*^compared to control in the inner area (O) for both doses of methimepip, *p* < 0.05.

The number of entries into the inner and central areas was significantly high in presence of an object than in the absence of an object in all groups (*p* < 0.02; Figure 1A). However, the duration of entries into the central area was significantly higher and the duration of entries into the outer area was significantly lower in the presence rather than in the absence of an object in all groups (*p* < 0.05) (Figure 1B).

In the absence of an object, there were no significant differences between groups (*p* > 0.10; Figure 1). However, in the presence of an object, mice treated with methimepip crossed significantly less into the inner (*p* < 0.05) and central area (*p* < 0.0002) and spent less time in the inner area (*p* < 0.05) compared to control (Figures 1A,B). There were no significant differences between the two doses of methimepip (*p* > 0.10; Figures 1A,B).

## Experiments 2 and 3—3D radial maze

### Materials and methods

#### Animals

Sixty four male Balb/c and 16 male C57BL/6J mice were purchased from Charles River Laboratories (Kent, UK). The animal weight was 25–28 g at the time of their arrival. They were housed four per cage. Individual mice could be identified by their cage number and their ear tags. They were left to acclimatize for 1 week before the start of the experiment. The colony room was held and animals were maintained as described in experiment 1.

#### Drug treatments

In experiment 2, there were two control groups (C57BL/6J, *n* = 8 and Balb/c, *n* = 8) which received physiological saline, and the other groups (*n* = 8 each) received a single injection of one dose each of methimepip (1, 2.5, and 5 mg/kg i.p.). In experiment 3, there were also two control groups (C57BL/6J, *n* = 8 and Balb/c, *n* = 8) which received physiological saline, and three Balb/c groups (*n* = 8 each) which received a single injection of one dose each of JNJ-5207852 (0.5, 1, and 5 mg/kg i.p) 30 min before introduction to the 3D maze. Methimepip and JNJ-5207852 were kind gifts from Professor Rob Leurs and Dr. Nicholas Curruthers (JNJ, USA), respectively. Selection of drug doses was based on previous *in vivo* studies (e.g., Kitbunnadaj et al., 2005; Jia et al., 2006).

#### Apparatus and testing procedure

The 3D maze is a modification of the classic radial-maze (Ennaceur et al., 2006). It is made from gray PVC (5 mm thick) and consists of eight arms radiating from a central platform. Each arm (51 × 11.2 cm) is made from two segments, extended from an octagonal shaped central hub (30 cm in diameter). The first segment of an arm (15.2 × 11.2 cm) is directly attached to the central platform and constitutes a bridge that allows access to the second segment (35 × 11.2 cm). Each bridge can be independently tilted upward or downward providing three maze configurations. In the present study, all bridges were tilted by 40° providing a configuration in which the arms are raised horizontal above the level of the central platform. Mice need to climb onto the bridges and then cross onto the arms. The floor of the bridges is covered with wire mesh. Sidewalls, about 1 cm high, extended the length of each bridge and arm. The end of each arm is extended with panels of identical size (20.2 × 11.2 cm) which are used to holding cues made of distinctive pattern drawings designed on plastic adhesive material and attached to a PVC board (18 × 11.2 cm). The maze is totally surrounded with a heavy beige-light colored curtain. The ambient light at the surface of the central platform is 180 Lux.

A mouse was removed from its cage, put in a small bucket in which it was weighted, and then tilted gently on the center platform of the maze. It was left to explore the test apparatus for 12 min. The surface of the maze was cleaned to minimize the effects of lingering olfactory cues. Any feces and urine were removed with paper towels, then cleaned with antibacterial solution followed by 90% ethanol and left to dry before the introduction of the next mouse.

#### Tools and recording measures

See similar section in experiment 1.

#### Measurement and statistical analysis

Differences among group means values for each measurement were tested for significance with One-Way ANOVA. Anything else as described in experiment 1.

### Results

**Figure 2:**
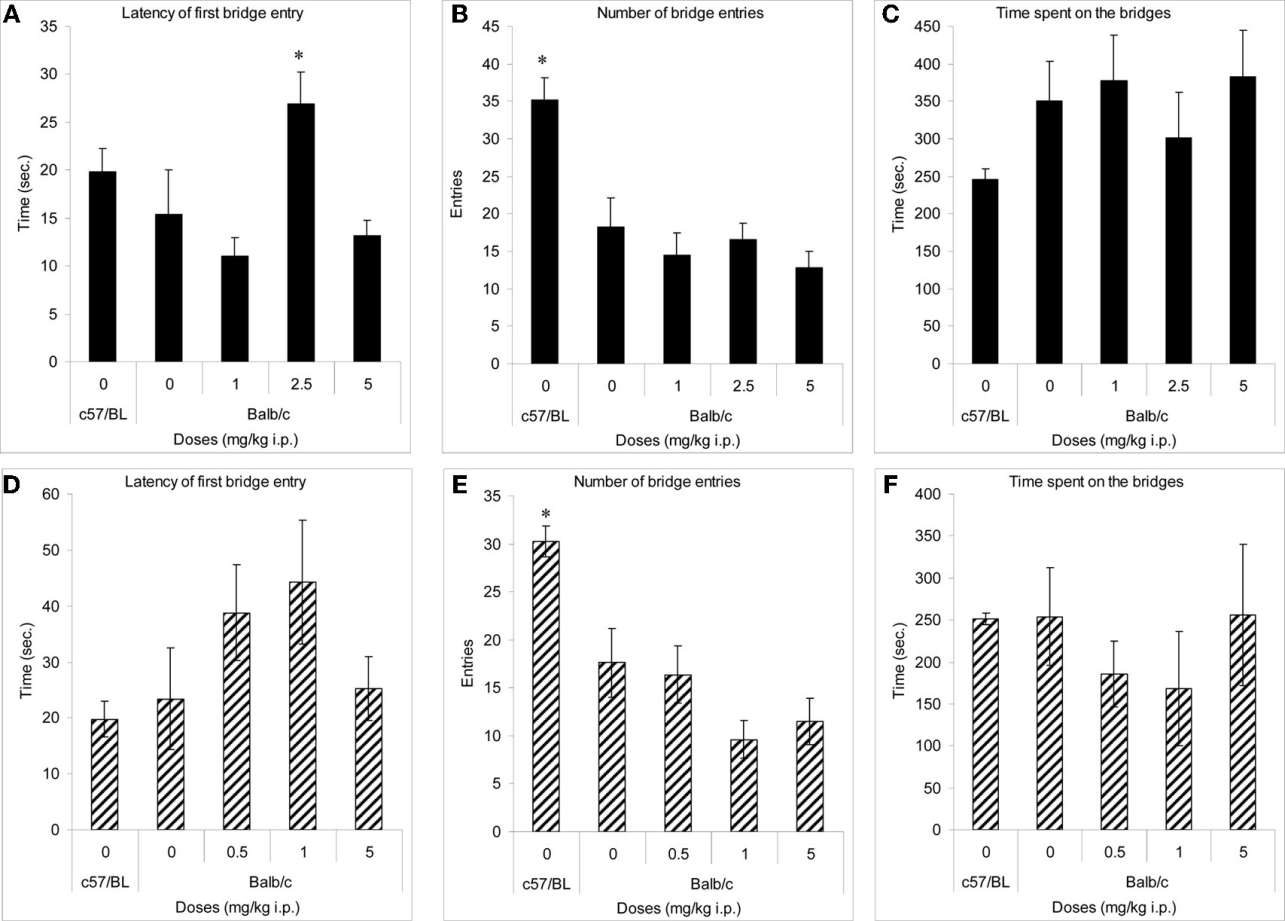
**Methimepip experiment with mice (A–C), JNJ-5207852 experiment with mice (D–F).** Mean (± s.e.m.) in the 3D-maze with mice. **(A)** Latency: ^*^compared to Balb/c saline and methimepip treated mice, *p* < 0.05; **(B)** number of entries, and **(E)** duration of entries: ^*^compared to Balb/c saline and all methimepip treated mice, *p* < 0.0002; (**C**, **D,** and **F**) no significant differences between groups, *p* > 0.10.

In both experiments, only C57BL/6J mice (comparator low anxiety strain) crossed onto the arms of the maze (not shown). Their number of crossings onto the bridges (Figures 2B,E) and duration of entries onto the bridges (Figures 2C,F) was significantly higher than in the other groups. Balb/c mice treated with methimepip (2.5 mg/kg i.p.) took significantly longer time to cross onto the bridges compared to Balb/c mice treated with saline (*p* < 0.02) and to Balb/c mice treated with methimepip at 1 and 5 mg/kg i.p. (*p* < 0.02; Figure 2A). There were no significant differences between Balb/c saline treated mice and either methimepip- or JNJ-5207852-treated mice in any other measures (Figures 2C–D and F).

## Discussion

We demonstrated in previous studies that C57BL/6J mice cross onto the distal arms of the 3D maze (Ennaceur et al., 2006; Ennaceur, 2011) and onto the steep hanging slopes of a novel elevated platform (e.g., Ennaceur et al., 2010; Michalikova et al., 2010). This demonstrates that these mice are able to take risks when exposed to unfamiliar open spaces (low anxiety strain). However, when a shelter is provided in the central platform of the maze or in the central area of the elevated platform, they stop crossing onto the arms and slopes, and spend most of the time inside the shelter. We have also confirmed this with rats in an enclosed and open space test with and without an object (Ennaceur et al., 2009a,b). The avoidance or preference responses observed in the presence of a shelter compare to those observed in the open-field, the plus-maze and the light/dark box. They do not provide unequivocal measures of fear-induced anxiety responses.

In the first experiment, using rats, the number of crossings into the central area of the open-field was significantly higher in the presence of an object than in the absence of an object in agreement with our earlier report (Ennaceur et al., 2009a,b). This avoidance of the empty central area cannot be attributed to a state of anxiety in animals. It is accounted for by animals' preference for walls and corners that form the open-field and also by the fact that there is nothing to encourage animals to stop and explore the central area (see Ennaceur et al., 2009a,b). In the open-field with an object, methimepip reduced the number of entries into and time spent in the inner and central areas. This not due to the effect of the drug on motor or exploratory activity as this is still higher than in saline and drug treated rats exposed to the open-field without an object. The presence of an object seems to further increase this avoidance response. This could be due to methimepip facilitating or exacerbating fear response when exposed to novelty. Indeed, it has been suggested that histaminergic neurotransmission in the brain is increased in stressful situations (Dere et al., 2010). In agreement, administration of histamine H_3_R agonists has been shown to increase the level of fear avoidance responses in fear conditioning paradigms (e.g., Baldi et al., 2005), in the plus-maze (Pérez-García et al., 1999). However, in a number of studies histamine H_3_R antagonists were also shown to increase the level of fear-induced avoidance responses in the plus-maze (Pérez-García et al., 1999; Bongers et al., 2004) and the light/dark box (Imaizumi and Onodera, 1993).

In order to evaluate the role of the H_3_R in anxiety, we adopted our recently developed and intensively characterized open space 3D maze test (Ennaceur et al., 2006, 2008; Ennaceur, 2011) with mice. In this test, animals are exposed to an open-space environment where the option to escape/avoid and explore are of equal valence. We reported that strains of mice display different levels of anxiety in this test, with Balb/c displaying consistently higher anxiety than C57BL/6J strains (Ennaceur et al., 2006, 2008; Ennaceur, 2011). Indeed, Balb/c mice alternate between the central platform and the proximal part of the arms (the bridges) and only C57BL/6J mice cross onto the distal part of the arms. In the present study, C57 mice crossed onto the arms of the maze which is in agreement with our previous findings (Ennaceur et al., 2006, 2008; Ennaceur, 2011) while all Balb/c treated with saline, methimepip or JNJ-5207852 did not cross onto the arms. These results do not suggest any effects of a selective H_3_ agonist and antagonist on measures of anxiety. This was confirmed (unpublished) using another selective H_3_ antagonist/inverse agonist, GSK334429B in a novel elevated platform open space test (Ennaceur, 2012). The differential effects of H_3_R modulation on avoidance and anxiety behaviors may be explained by the growing anatomical and functional evidence for H_3_ auto- and hetero-receptor heterogeneity (Giannoni et al., 2009, 2010; Passani and Blandina, 2011).

In most studies, the effects of different doses of a drug treatment are compared to saline as control. In experiments 2 and 3, we included C57BL/6J as a second control group. This is simply because in order to assess the anxiolytic effect of a drug one would have to choose the strain of animals with high anxiety and if the drug produces a reduction in anxiety one would need to demonstrate that this reduces anxiety below or at least to the level of low-anxiety strains of animals.

As stated in our introduction, avoidance of the central area or an object in the central area may be indicative of fear response but also a preference response of walls and corners. If the definition of anxiety is based on the conflict between the drive to explore and the drive to avoid, one must demonstrate evidence in animals of the drive to explore in this test. In the 3D maze, all parts of the maze are open and unprotected. When released on the central platform, animals explore the bridges but do not venture further onto the arms; they alternate between the bridges and the central platform. The drive to explore is clearly evident by the crossings onto the bridges.

In previous studies, we demonstrated the difference between test conditions that promotes fear-induced escape/avoidance and fear-induced anxiety (Ennaceur et al., 2006; Michalikova et al., 2010; Ennaceur, 2012). When exposed for the first time to a 3D-maze, C57BL/6J mice venture onto the arms of the maze while Balb/c mice explore only the central platform and the bridges. However, if the central platform is enclosed they behave like Balb/c mice; they do not cross onto the arms of the maze. Comparable behavior was observed in the elevated platform with slopes. C57BL/6J mice stop crossing onto the slopes when a hiding place is provided in the middle of the platform (Michalikova et al., 2010; Ennaceur, 2012). In the presence of a protected space, animals may not feel the need to take risks away from the protected space; this is the case in the current unconditioned tests of anxiety for rats and mice.

In summary, the results of our present experiments with selective agonist and antagonist drugs provide new evidence that the H_3_R may have a role in fear-induced avoidance responses, but not in anxiety.

### Conflict of interest statement

The authors declare that the research was conducted in the absence of any commercial or financial relationships that could be construed as a potential conflict of interest.
